# Cross-organ microbes modulate host upper gastrointestinal health and disease via the oral-gastric microbiome axis

**DOI:** 10.3389/fcimb.2026.1941478

**Published:** 2026-09-29

**Authors:** Xizhen Peng, Wei Peng, Yu Zou, Zhaodong Li

**Affiliations:** 1 Department of Gastroenterology, Clinical Medical College and the First Affiliated Hospital of Chengdu Medical College, Chengdu, Sichuan, China; 2 Key Laboratory of Digestive Microecology Medicine of Sichuan Health Commission, Chengdu, Sichuan, China; 3 Key Laboratory of Digestive System Tumors and Microenvironment of Sichuan Provincial University, Chengdu, Sichuan, China; 4 Department of Gastroenterology, Mianyang Central Hospital, Mianyang, Sichuan, China

**Keywords:** cross-organ microbes, gastric diseases, oral microbiota, oral-gastric axis, translocation

## Abstract

The human body harbors highly diverse and complex microbial communities across various regions. Among these, the oral cavity and stomach are directly connected and engage in dynamic microbial interactions. Microorganisms capable of migrating between these two distinct niches are referred to as trans-organ microbes, which constitute a core component of the oral–gastric microbiome axis. In this review, we elucidate the mechanisms underlying microbial translocation along the oral-gastric axis. We further discuss how these cross-organ microbes exert bidirectional influences on health and disease in upper gastrointestinal environments. They contribute to mucosal homeostasis and modulate immune responses, yet under certain conditions, they can also promote pathologies such as gastritis, gastric cancer, and oral disorders. The migration and colonization patterns of microbes across organs also hold potential for developing diagnostic biomarkers, as well as for designing innovative microbiome-based interventions targeting the upper gastrointestinal tract. In summary, this review highlights the oral-gastric microbiome axis as a critical frontier for understanding and manipulating microbiome dynamics in human health and disease.

## 1 Introduction

A diverse microbial community, made up of trillions of microorganisms, colonize the human body and its different habitats. These microbial communities have profound impacts on the host by activating the immune system, defending against pathogens, and can serve as triggers and drivers of chronic disease processes (Gilbert et al., 2018). The oral cavity serves as the entry point of the human digestive tract and represents a major reservoir of microbiota, which plays a significant role in upper gastrointestinal health and disease (Wade, 2013; Kunath et al., 2024). The interaction between oral microorganisms and gastric microorganisms and their pathogenic mechanisms are becoming a research hotspot. Oral pathogenic bacteria can transfer to the stomach through swallowing or co-aggregation, and damage the gastric mucosal barrier, promoting the occurrence of gastritis, gastric ulcers and gastric cancer (Chen et al., 2022; Xia et al., 2025). Conversely, gastric microorganisms can also influence oral health. For instance, *Helicobacter pylori* (*H. pylori*) can colonize dental plaque and cavities, forming a symbiotic biofilm with *Streptococcus mutans* (*S. mutans*) that exacerbates dental caries and periodontal disease (Zhang et al., 2018). In patients with gastroesophageal reflux disease, stomach acid entering the oral cavity can alter salivary pH. This change inhibits periodontal pathogens such as *Porphyromonas* while promoting the growth of acid-tolerant bacteria like *Streptococcus*, ultimately disrupting the oral microecosystem equilibrium (Kawar et al., 2021). The bidirectional exchange of microorganisms between the oral cavity and the stomach can reshape the microbial ecosystems of these two parts, forming oral–gastric microbiome axis (**Figure 1**).

**Figure 1:**
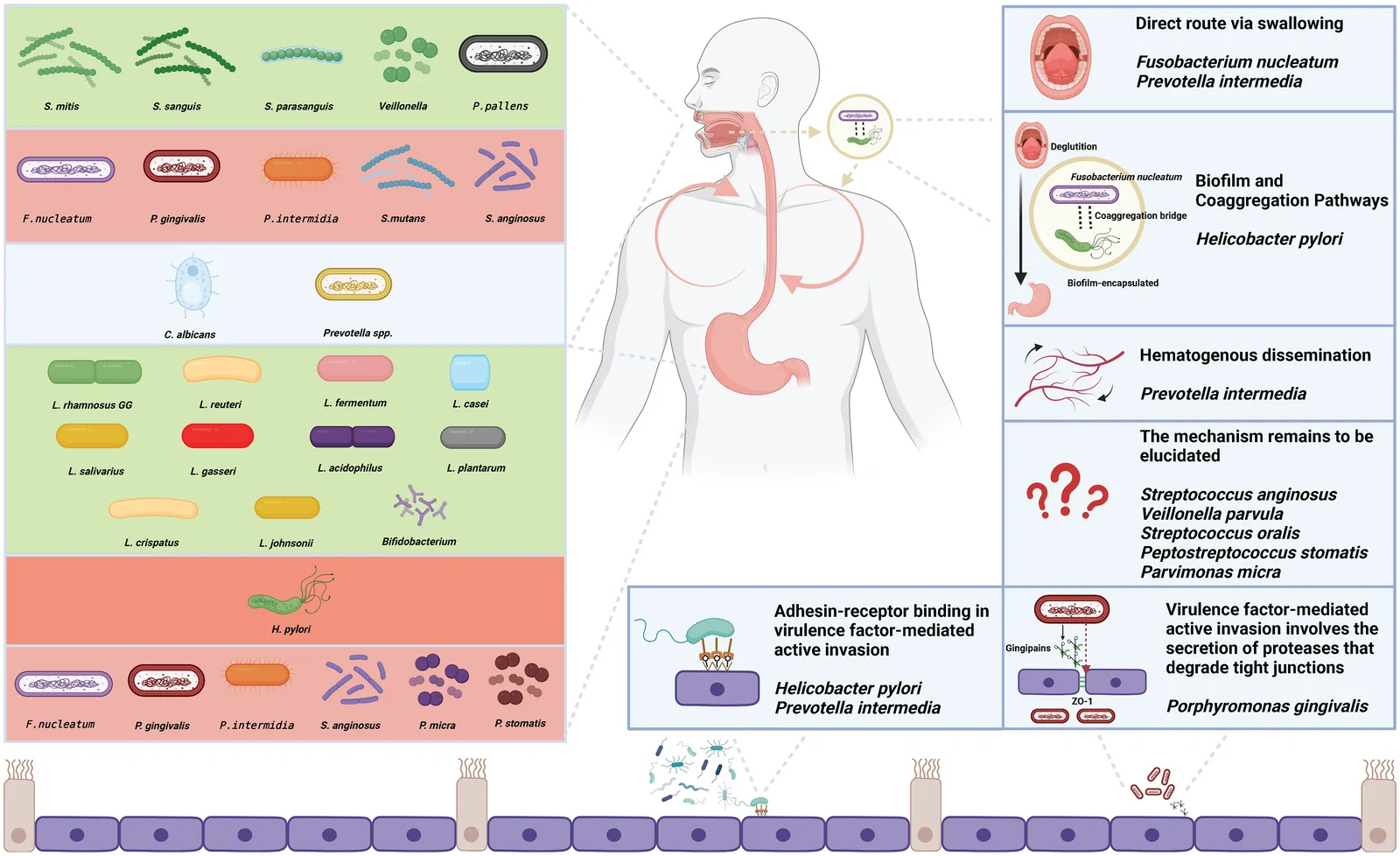
The oral–gastric microbiome axis: bidirectional microbial exchange and translocation mechanisms. The oral microbiota comprises diverse bacterial and fungal species. These microorganisms can translocate to the stomach via multiple routes: direct swallowing, biofilm/coaggregation pathways, hematogenous dissemination, and virulence factor-mediated active invasion (including adhesin-receptor binding and secretion of proteases that degrade tight junctions). Key oral and gastric microbial taxa involved in this axis are listed in the figure, with arrows indicating their potential routes of transfer.

## 2 Oral microbiota: composition and its role in oral health and disease

### 2.1 Composition of the oral microbiota

The oral microbiome is the second-largest microbial ecosystem in the human body after the gut. More than 700 bacterial species have been identified, spanning nine major phyla (*Firmicutes*, *Bacteroidetes*, *Proteobacteria*, *Actinobacteria*, *Fusobacteria*, *Spirochaetes*, etc.), together with fungi (mainly *Candida*), archaea (*methanogens*) and a rich virome of bacteriophages (Dewhirst et al., 2010; Wade, 2013). The community can be grouped as follows: (1) Beneficial symbionts:*Streptococcus salivarius* has been shown to reduce the incidence of dental black stains, plaque, and tooth decay in children. It also decreases the occurrence of pharyngeal and recurrent streptococcal diseases (Al-Akel et al., 2024). *Veillonella* spp. are obligate anaerobes that convert *Streptococcal lactate* into the weaker acid propionate, buffering enamel demineralization and stabilizing dental-plaque homeostasis (Mashima and Nakazawa, 2015). *Rothia* and *Corynebacterium* colonize mucosal surfaces early, form a reticular matrix and create microoxic-to-anaerobic gradients that facilitate biofilm maturation and later colonization by strict anaerobes (Welch et al., 2016). (2) Opportunistic pathogenic bacteria (those enriched during dysbiosis): *S. mutans* and *Lactobacillus* have strong acid-producing and acid-tolerant properties, which drive enamel demineralization and are primary pathogens for dental caries (Takahashi and Nyvad, 2011). *Porphyromonas gingivalis* (*P. gingivalis*) produces gingipain proteases and lipopolysaccharides that impair neutrophil phagocytosis and enhance bacterial survival within these cells (Ruan et al., 2023). *Fusobacterium nucleatum* (*F. nucleatum*), as a bridge between early Gram-positive colonizing bacteria and late anaerobic pathogenic bacteria, promotes interspecies aggregation through co-aggregation, assisting strict anaerobic bacteria in surviving in oxygen-rich environments, thereby promoting the maturation of biofilms and dysbiosis (Bradshaw et al., 1998).

### 2.2 The impact of oral microbiota on oral diseases and its mechanisms

The oral microbiota is implicated in a range of oral conditions, including periodontal disease, dental caries and oral cancer (Baker et al., 2024). Periodontal disease, a chronic infectious disease affecting the supporting tissues of the teeth (including the gingiva, periodontal ligament, and alveolar bone), arises from a disruption of host–microbial homeostasis within the periodontal pocket (Hajishengallis and Chavakis, 2021). Under conditions of oral health, the inflammatory response and the periodontal microbiome maintain a dynamic bidirectional equilibrium. In periodontitis, this balance is disrupted: Gram-negative bacteria proliferate extensively and ultimately displace the microbial communities associated with periodontal health (Holt and Ebersole, 2005). Among the enriched species are the classically recognized red-complex triad—comprising *Treponema denticola*, *P. gingivalis* and *Tannerella forsythia* —as well as other organisms such as *Filifactor alocis*, *Peptoanaerobacter stomatis*, and *Saccharibacteria*. Together, these microbial groups constitute the principal members of the periodontitis-associated microbial community (Curtis et al., 2020). Furthermore, proliferation of acidogenic and acid-tolerant species—such as *S. mutans*, *lactobacilli*, *Veillonella*, *Candida albicans*, *Epstein-Barr virus*, and *Prevotella* species also drive biofilm formation and ecological changes (Benseddik et al., 2025).

Dental caries is an infectious, biofilm-mediated disease of the calcified tooth tissues, driven by acid production from bacterial fermentation of dietary carbohydrates. The disease process is characterized by phasic demineralization and remineralization; however, once cavitation occurs, the resultant structural damage is irreversible (Kutsch, 2014; Dayo et al., 2021). When the oral microecology is imbalanced, the cariogenic bacteria will dominate. The most prevalent cariogenic species include oral streptococci, notably *S. mutans*, and *Lactobacilli* (Szkaradkiewicz-Karpinska and Szkaradkiewicz, 2021). *S. mutans* is considered a primary pathogen in dental caries due to its ability to synthesize extracellular polysaccharides via glucosyltransferases, form biofilm matrices, and promote tooth demineralization through acid production coupled with inherent acid resistance (Ucuncu et al., 2025). Similarly, *Lactobacillus acidophilus* (*L. acidophilus*) exhibits strong acidogenic and acid-tolerant properties. It also produces extracellular polysaccharides that facilitate bacterial adhesion to enamel and dental plaque formation, ultimately contributing to tooth decay (Aas et al., 2008). Beyond these, other bacteria are associated with specific caries phenotypes. For example, *Actinomyces naeslundii* is closely linked to the development of root caries (Bergeron et al., 2000); *Scardovia wiggsiae* has been found to accumulate significantly in cases of severe early childhood caries (Kressirer et al., 2017).

Oral squamous cell carcinoma (OSCC), the most prevalent head and neck cancer, constitutes about 90% of oral malignancies and impairs appearance, speech, swallowing, and taste perception (Tan et al., 2023). The significant role of tumor microenvironment in the initiation, development, and progression of OSCC has been widely recognized. A diverse range of oral microorganisms—including bacteria such as *P. gingivalis* and *F. nucleatum*, viruses like HPV and EBV, and fungi such as *Candida albicans* —have been linked to the development and progression of OSCC (Pignatelli et al., 2023; Starska-Kowarska, 2025). In addition, *Parvimonas micra*, which is enriched in OSCC tissues, interacts with the tumor cell receptor CKAP4 via its surface protein TmpC, activating HIF-1α-driven glycolysis and autophagy to facilitate epithelial–mesenchymal transition and lymph node metastasis (Qi et al., 2025). Moreover, emerging evidence has associated *Fusobacterium periodonticum*, *Haemophilus influenzae*, and *Filifactor alocis* with the onset and progression of OSCC (Stasiewicz and Karpiński, 2022).

Furthermore, oral lichen planus and Behçet’s syndrome have also been confirmed to be associated with oral microbiota. *H. pylori* infection is closely related to erosive oral lichen planus, and the level of volatile sulfur compounds in patients with oral lichen planus is significantly higher than that in healthy individuals (Guan et al., 2024). In patients with Behçet’s syndrome, the *H. pylori* infection rate is higher, and symptoms such as oral ulcers, genital ulcers, and skin lesions can be improved after eradication therapy (Avci et al., 1999).

## 3 Gastric microbiota: composition, disease associations, and mechanistic insights

### 3.1 Composition of the gastric microbiota

The stomach’s harsh acidic conditions and robust peristaltic activity led to the long-held belief that it was a sterile organ. The 1983 discovery of *H. pylori* within the stomach revealed that the gastric environment is indeed capable of being colonized by microorganisms (Marshall and Warren, 1984). Using bacterial culture method, bacteria such as *Clostridium sp*, *Lactobacillus sp* and *Veillonella sp* have been found to exist in the healthy human stomach (Zilberstein et al., 2007). With the advancement of sequencing technologies, particularly high-throughput sequencing based on the 16S rRNA gene, it has been found that the dominant bacterial phyla in the stomach under healthy conditions include *Actinobacteria*, *Bacteroidetes*, *Firmicutes*, and *Proteobacteria* (including *H. pylori*) (Ianiro et al., 2015). Furthermore, *Tsuda* et al. performed a meta-16S analysis of the gastric luminal microbiome, they identified *Streptococcus* and *Prevotella* as the most prevalent genera, accounting for approximately 50% of all species detected in the stomach (Tsuda et al., 2015).

Under specific pathological conditions, the composition of the gastric microbiota undergoes significant alterations. For instance, patients with bile reflux exhibit elevated bacterial diversity in the gastric environment. Genera such as *Comamonas*, *Halomonas*, *Bradymonas*, *Pseudomonas*, *Marinobacter*, *Arthrobacter*, and *Shewanella* are significantly enriched in these individuals (Huang et al., 2022). Additionally, dietary factors can also affect the composition of the gastric microbiota (Paul et al., 2022). Meanwhile, the microbes in the stomach interact with each other, and multiple studies have shown that *H. pylori* infection can affect the abundance of gastric microbiota (Chen et al., 2021; Wizenty and Sigal, 2025). On the other hand, certain gastric commensal bacteria demonstrate potent anti-*H. pylori* properties, such as *Lactobacillus johnsonii*, *Lactobacillus murinus*, *Lactobacillus reuteri* (*L. reuteri*), and *Streptococcus mitis* (*S. mitis*), which may reduce the pathogenicity of *H. pylori* (Zaman et al., 2014; Khosravi et al., 2016).

### 3.2 The impact of gastric microbiota on gastric diseases and its mechanisms

Recent evidence suggests that gastric microbiota may contribute to the pathophysiology of various upper gastrointestinal diseases. Among these, gastric cancer is the fifth most common malignancies worldwide and the third most fatal, with over one million new cases reported annually (Lu et al., 2022). *H. pylori* infection is an independent risk factor for gastric cancer. The underlying molecular mechanisms are thought to involve dysregulation of the STAT3, NF-κB, Hippo, and Wnt/β-catenin signaling pathways. Moreover, *H. pylori* can trigger oxidative stress, DNA damage, chronic inflammation, and apoptosis processes that collectively facilitate carcinogenesis (Duan et al., 2025). Furthermore, some non-*H. pylori* gastric microbes have also been confirmed to be correlated with the development of gastric cancer, influencing different types of gastric cancer respectively. For example, the *phyla Fusobacteria*, *Bacteroidetes*, and *Patescibacteria* were increased in signet-ring cell carcinoma, whereas *Proteobacteria* and *Acidobacteria* are mainly identified in adenocarcinoma (Wang et al., 2023).

While *H. pylori* is established as the primary bacterial cause of gastritis, it is not the sole contributor to the disease. Recent studies on *H. pylori-*negative gastritis have shown that in the gastric mucosa of gastritis patients, the relative abundances of bacterial families such as *Micrococcaceae*, *Streptococcaceae*, and *Leuconostocaceae*, as well as genera including *Streptococcus*, *Weissella*, and *Cryptobacterium*, are significantly higher compared to non-gastritis groups (Martinez-Montoro et al., 2025). These findings suggest that these microbial communities may also play an important role in the pathogenesis of gastritis. Furthermore, in a study conducted in China, in addition to *H. pylori*, the main pathogens causing gastritis include *Methylobacillus* and *Arthrobacter* (Coker et al., 2018).

Autoimmune atrophic gastritis (AAG) is an organ-specific immune-mediated condition characterized by a progressive loss of acid-secreting parietal cells leading to hypo-achlorhydria. A study on the human gastric microbiota reported that at the genus level, after *Streptococcus*, *Gemella* and *Haemophilus* were the most represented genera in the AAG patients (Parsons et al., 2017). Interestingly, *Giulia* et al., using 16S rRNA sequencing, showed that women with healthy stomachs had higher gastric bacterial abundance and lower microbial diversity compared to men. However, due to hypochlorhydria and the non-acid intragastric environment, AAG appears to reset gender differences in gastric bacterial abundance and reduce biodiversity in males, demonstrating a greater extent of dysbiosis in terms of reduced biodiversity in men (Pivetta et al., 2023).

## 4 Formation and dynamic changes of the oral-gastric microbiome axis

### 4.1 The cross-organ translocation of microorganisms serves as the foundation for the formation of the oral-gastric microbiome axis

Cross-organ microbes refer to microbial communities that transcend single-organ boundaries, exhibiting dynamic interactions across different anatomical sites (Tang et al., 2025; Chen et al., 2026). Unlike microorganisms that undergo transient passage (i.e., sporadic detection without evidence of viability), the cross-organ microbes defined in this review refer to those that exhibit active colonization, as supported by repeated detection, culture- or RNA-based evidence of viability, or demonstrable functional effects on the gastric mucosa. These microbes not only colonize their primary ecological niches but also actively migrate to distant organs or exert functional modulation on them through metabolic byproducts, immune regulation, and neural signaling pathways. In addition, there exists another group of microorganisms that can translocate from the oral cavity to the stomach and persist there, influencing the gastric microenvironment, yet their specific pathogenic mechanisms and colonization routes remain unclear. We regard these as putative trans-organ microbes, and have listed and described them in **Tables 1**, **2** (Parsons et al., 2017; Xia et al., 2025). The oral and gastric environments are continuously connected through saliva swallowing and food intake, forming a natural microbial communication channel. Multi-omics evidence has revealed that the oral microbiome is the primary source of gastric microbes (Xia et al., 2025). Oral microbes reach the stomach through daily swallowing, and shifts in their community correlate with gastric precancerous lesions and cancer risk (Liu et al., 2024). In addition, reduced gastric acidity (particularly due to proton pump inhibitor use) may shift the gastric microbial composition toward an oral cavity-like profile (Hu et al., 2025).

**Table 1:** Major cross-organ microbes with promoting effects on gastric health.

Name of bacterial genus	Name of bacterial species	Key mechanisms related to gastric health	Refs.
*Streptococcus*	*Streptococcus mitis*	Produce hydrogen peroxide to combat gastric cancer cells and induce their death.	(Yang et al., 2025)
	*Streptococcus parasanguinis*	Inhibits the growth and biofilm formation of pathogenic bacteria by reacting hydrogen peroxide with nitrite to generate reactive nitrogen species.	(Baty et al., 2022)
*Lactobacillus*	*Lactobacillus rhamnosus GG*	Survives gastric acid/bile; adheres to enterocytes; produces lectin-like proteins inhibiting pathogens; modulates immune response.	(Segers and Lebeer, 2014; Capurso, 2019)
	*Lactobacillus acidophilus and Lactobacillus plantarum*	Reduces *H. pylori* adhesion; inhibits *H. pylori*-induced gastric inflammation.	(Shen et al., 2023)
	*Lactobacillus crispatus*	Modulates inflammation by regulating pro- and anti-inflammatory cytokine expression.	(Fakharian et al., 2023)
	*Lactobacillus reuteri*	Produces reuterin inhibiting *H. pylori*; modulates immune pathways to reduce inflammation.	(Garcia-Castillo et al., 2019; Lee et al., 2025)
	*Lactobacillus fermentum*	Regulates cytokine levels to enhance resistance to *H. Pylori* infection.	(Garcia-Castillo et al., 2020)
*Bifidobacterium*	*Bifidobacterium longum* and *Bifidobacterium bifidum*	Inhibit *H. pylori* colonization via competitive exclusion, organic acid production; enhance IgA secretion, strengthen the mucosal immune barrier, and block the migration and colonization of oral pathogenic bacteria to the stomach.	(Kaci et al., 2014; Gao et al., 2015; Chen et al., 2021)
Veillonella*	NA	Convert lactic acid into less acidic propionic acid.	(Mashima and Nakazawa, 2015)
*Prevotella*	*Prevotella pallens*	Produce short-chain fatty acids such as propionic acid through the fermentation of dietary fibers to exert anti-inflammatory effects.	(Han et al., 2019; Han and Wang, 2026)

NA, Not available;*H. pylori, Helicobacter pylori;* *Veillonella have been detected in the healthy human stomach via bacterial culture. However, while their proposed gastric protective effects are inferred from oral metabolic activities and salivary transit, direct evidence of viable colonization and functional regulation within the gastric mucosa remains lacking. Consequently, they are designated as putative trans-organ microbes in **Table 1**.

**Table 2:** Major cross-organ microbes involved in the pathogenesis of gastric diseases.

Name of microbes	Mechanisms of traversing the oral mucosal barrier	Types of gastric diseases	Refs.
*Helicobacter pylori*	Co-aggregation and biofilms; Endosymbiotic protection; Symbiotic metabolism and adhesion; Adhesin–receptor interactions	Chronic gastritis; Peptic ulcers; Gastric cancer; Mucosa-associated lymphoid tissue lymphoma	(Magalhães et al., 2016; Park et al., 2016; Sánchez-Alonzo et al., 2020; Lupu et al., 2025)
*Fusobacterium nucleatum*	Direct saliva swallowing to stomach	Gastric cancer	(Pignatelli et al., 2023; Wang et al., 2024; Zheng et al., 2025)
*Porphyromonas gingivalis*	Secrete gingipains to degrade the tight junction proteins between cells; Actively cross the mucosal barrier through transmigration between epithelial cells; Phagocytosed by the host cell	Chronic atrophic gastritis; Intestinal metaplasia; Gastric cancer	(de Jongh et al., 2023; Ruan et al., 2023; Muñoz-Medel et al., 2024; Lin et al., 2025)
Streptococcus anginosus *	NA	Gastric cancer	(Fu et al., 2024; Guo et al., 2024; Xia et al., 2025; Zhou et al., 2026)
*Prevotella intermedia*	Hematogenous spread from oral cavity; Direct saliva swallowing to stomach; Virulence factor-mediated translocation.	Chronic non-atrophic gastritis; Chronic atrophic gastritis; Intestinal metaplasia; Dysplasia; Gastric cancer	(Yamanaka et al., 2011; Kononen and Gursoy, 2021; Park et al., 2021; Aitmanaite et al., 2023; Chen et al., 2023; Liang et al., 2024)
Veillonella parvula *	NA	Gastric cancer	(Lei et al., 2024)
Streptococcus oralis *	NA	Gastric cancer	(Lei et al., 2024)
Peptostreptococcus stomatis *	NA	Gastric cancer	(Coker et al., 2018)
Parvimonas micra *	NA	Gastric cancer	(Coker et al., 2018)

NA, Not available; *Studies have confirmed that methionine derived from *Streptococcus anginosus* promotes gastric cancer progression; *Veillonella parvula* and *Streptococcus oralis* form a unique oral-associated microbiota in gastric cancer tissues (particularly in the upper third of the stomach), modifying the tumor microenvironment pH by producing lactic acid and short-chain fatty acids, thereby promoting tumor growth and metastasis; *Peptostreptococcus stomatis* and *Parvimonas micra* have also been confirmed to occupy a central position in the gastric cancer ecological network. However, the specific mechanisms by which these microorganisms traverse the oral mucosal barrier remain unclear; accordingly, we define these microorganisms as putative cross-organ microbes.

Taking *H. pylori* as an example, we elaborate on its translocation mechanism in detail: (1) Co-aggregation and biofilms-periodontal pathogens such as *F. nucleatum* and *P. gingivalis* co-aggregate with *H. pylori*, forming mixed biofilms that help the bacterium tolerate gastric acid and migrate along the oral–gastric axis (Park et al., 2016). (2) Endosymbiotic protection - *H. pylori* can reside inside oral commensals like *Candida albicans*, gaining resistance to desiccation, antibiotics and heat and thus enhancing survival during transit from mouth to stomach (Sánchez-Alonzo et al., 2020). (3) Symbiotic metabolism and adhesion – co-culture with oral *S. mutans* increases organic-acid and mutacin production, creating microaerophilic, low-pH niches that favor *H. pylori* attachment and biofilm maturation (Lupu et al., 2025). (4) Adhesin–receptor interactions – *H. pylori* BabA and SabA bind salivary MUC5B and gastric MUC5AC, while NapA recognizes sulfated oligosaccharides in saliva, promoting oral colonization and subsequent gastric entry (Magalhães et al., 2016).

Accumulating evidence along the oral–gut microbiome axis indicates that gut dysbiosis can significantly reshape the oral microbial ecosystem (Khanna et al., 2026). Given that the stomach anatomically bridges the oral cavity and the intestine, it is plausible that gastric colonizers may exert analogous modulatory effects on oral microbial homeostasis via retrograde translocation to the upper aerodigestive tract. However, the presence of the esophagogastric barrier limits the retrograde migration of bacteria from the gastrointestinal tract to the oral cavity. Such migration primarily occurs in pathological processes such as vomiting, gastroesophageal reflux (GER), and certain gastrointestinal motility disorders (Yucel, 2019). GER is the most direct route by which gastric bacteria enter the esophagus and oral cavity (Wang, 2024). In affected patients, lower esophageal sphincter incompetence leads to frequent reflux of gastric contents (including gastric fluid and the bacteria it carries) into the distal esophagus (Yao et al., 2026). Studies have shown that esophageal microbiota in patients with reflux esophagitis and Barrett’s esophagus exhibits a significantly higher proportion of Gram-negative bacteria, along with a decreased abundance of the otherwise predominant Streptococcus genus - a compositional shift that is closely associated with the reflux input of gastric microbiota (Zou et al., 2023). Furthermore, in patients with GER and Barrett’s esophagus, frequent gastric acid reflux can transiently alter local oral pH, suppressing the growth of certain acid-sensitive streptococci while conferring a selective advantage to acid-tolerant Gram-negative bacteria, resulting in a decreased abundance of Streptococcus and a markedly increased proportion of Gram-negative bacteria in the oral microbiota (Yang et al., 2009).

Interestingly, studies in animal models have observed that *Lactobacillus murinus* can colonize both gastric and esophageal epithelia in a biofilm-like manner, suggesting that some gastric bacteria may establish ecological niches in the esophagus and oral cavity through active colonization rather than passive reflux (Almirón et al., 2013). Clinical observations have also revealed that the relative abundances of *Klebsiella pneumoniae* and *Prevotella copri* in the esophageal microbiota of patients with Barrett’s esophagus are positively correlated with their levels in the gastric microbiota, suggesting microbial exchange between the two sites (Lopetuso et al., 2020).

### 4.2 Microbial metabolites are a vital component of the oral-gastric microbiome axis

Microbial metabolites function as key mediators in the oral-gastric axis. The metabolites of tryptophan produced by oral microorganisms play a crucial role in maintaining the homeostasis of the digestive tract. The commensal bacteria in the oral cavity and the gastrointestinal tract, such as *Lactobacillus species*, *Bacteroides species*, and *Clostridium species*, can convert tryptophan into various indole derivatives, including indole-3-lactic acid (ILA), indole-3-propionic acid (IPA), and indole-3-aldehyde (IAld), etc (Hou et al., 2023). These metabolites act as endogenous ligands for the aryl hydrocarbon receptor (AhR), activating the AhR signaling pathway, inhibiting IL-17/Th17-related inflammatory responses, enhancing the function of CD8+ T cells, and inhibiting excessive proliferation of epithelial cells, thereby exerting anti-tumor effects (He et al., 2026). It is noteworthy that although pathogenic oral bacteria such as *F. nucleatum* can also produce indole-3-acetic acid, in gastric cancer tissues, their metabolic product spectrum is closely associated with gastric carcinogenesis, suggesting that the metabolites of tryptophan may have certain environmental dependence and a bidirectional effect (Nie et al., 2025).

Bile acid metabolites are also key effector molecules of the oral-gastric microbiome axis. Oral bacteria (such as *P. gingivalis*) participate in the dissociation and modification of primary bile acids, generating secondary bile acids such as deoxycholic acid (Li et al., 2022). These metabolites regulate energy metabolism and inflammatory responses by activating Farnesoid X receptor and G-protein-coupled bile acid receptor. However, choline-derived metabolites such as Trimethylamine N-oxide have a pro-tumor effect, amplifying oxidative stress, activating the NF-κB signaling pathway, and disrupting the integrity of epithelial tight junctions, creating a favorable microenvironment for cancer progression (He et al., 2026). These findings reveal the dual roles of microbial metabolites in tumor development: there are protective metabolites such as indole derivatives, and there are also carcinogenic metabolites such as Trimethylamine N-oxide, which interact with inflammatory, genotoxic, and immune pathways to collectively promote gastric carcinogenesis.

Short-chain fatty acids (SCFAs) also function as systemic immunomodulatory and anti-inflammatory agents (Kerstens et al., 2024). In the oral cavity, specific metabolic pathways carried out by microorganisms contribute to SCFA production. For instance, *Takahashi* et al. demonstrated that oral bacteria such as *Prevotella* and *Porphyromonas* can break down proteins and peptides into amino acids, which are further metabolized to generate SCFAs (Takahashi, 2015). These metabolites play a significant role in supporting local oral health and broader metabolic processes. Moreover, *Huang* et al. found that SCFAs produced by oral bacteria exhibit direct antibacterial activity, inhibiting the growth of pathogens such as Shigella flexneri and thereby helping to maintain the balance of the oral microbiota (Huang et al., 2011).Metabolites derived from the oral-gastric microbiome axis exert profound regulatory effects on oral microecology. Lipopolysaccharide released by Gram-negative bacteria in the refluxate can activate the Toll-like receptor 4 (TLR4)/NF-κB signaling pathway in oral epithelial cells, promoting the secretion of proinflammatory cytokines such as IL-8 and IL-1β, thus forming a vicious cycle of reflux-inflammation-dysbiosis that further exacerbates oral microecological disorders (Yáñez et al., 2024). Ghrelin, a peptide hormone primarily secreted by the gastric mucosa, is not only involved in the regulation of appetite and the release of growth hormone, but also exerts significant anti-inflammatory and immunomodulatory effects (Słotwińska, 2020). In the oral cavity, ghrelin and its receptor are widely expressed in salivary glands, taste buds of the lingual epithelium, oral mucosal epithelium, and gingival fibroblasts. Ghrelin levels comparable to or even higher than those in plasma can be detected in both saliva and gingival crevicular fluid (Ohta et al., 2011). These locally derived ghrelin molecules play an important role in maintaining oral mucosal homeostasis and regulating innate immune responses. Furthermore, *Warzecha* et al. found in a rat model that exogenous ghrelin significantly reduced the concentration of the proinflammatory cytokine IL-1β in the oral mucosa and accelerated the healing of oral ulcers (Warzecha et al., 2013). Moreover, ghrelin could inhibit the expression of proinflammatory factors such as CCL2 and IL-6 in human gingival fibroblasts induced by *F. nucleatum*, suggesting that gastric-derived hormones may exert a protective regulatory effect on oral inflammation (Nogueira et al., 2022).

## 5 Core mechanisms by which the oral-gastric microbiome axis promotes host gastric health

The oral-gastric microbiome axis is an important physiological mechanism for maintaining the homeostasis of the upper digestive tract, which plays a crucial role in the development and maintenance of the mucosal immune system. Studies indicate that beneficial oral bacteria such as *Streptococcus* and *Lactobacillus* can translocate to the stomach via the oral-gastric microbiome axis, promoting gastric health through mechanisms including the production of antimicrobial substances and modulation of local immune responses (**Table 1**).

### 5.1 Production of antimicrobial substances that can directly inhibit pathogens

Oral symbiotic bacteria can synthesize various antimicrobial effector molecules, forming the first chemical barrier against pathogenic microorganisms. *S. mitis* is one of the earliest colonizing commensal bacteria in the oral cavity and gastrointestinal tract. It can produce hydrogen peroxide to combat cancer cells and induce their death; in a gastric cancer model, oral administration of *S. mitis* significantly inhibits the growth of tumors and prolongs survival, and the mechanism is related to the inhibition of M2-type macrophage polarization, thereby reshaping the tumor immune microenvironment (Yang et al., 2025). *Streptococcus parasanguinis* inhibits the growth and biofilm formation of pathogenic bacteria such as *S. mutans*, *Aggregatibacter actinomycetemcomitans*, and *Enterococcus faecalis* by reacting hydrogen peroxide with nitrite to generate reactive nitrogen species (such as peroxynitrite) (Baty et al., 2022). *L. reuteri* produces reuterin, which directly inhibits the growth of *H. pylori* (Garcia-Castillo et al., 2019), and this bacterium can survive in gastric acid and colonize the gastric mucosa (Garcia-Castillo et al., 2020; Ivashkin et al., 2024).

### 5.2 Competition exclusion and acidity regulation

Probiotics inhibit the adhesion and colonization of pathogenic bacteria through space-occupying effects and organic acid metabolism. *Bifidobacterium* supplementation enables the host to inhibit *H. pylori* colonization via competitive exclusion and organic acid (acetic and lactic acid) production, thereby improving gastric mucosal barrier function (Chen et al., 2021). *L. acidophilus* and *Lactobacillus plantarum* could reduce the adhesion of *H. pylori* and inhibit the gastric inflammatory response induced by *H. pylori* infection (Shen et al., 2023). *Veillonella*, as one of the core symbiotic bacterial communities in the early colonization of the oral cavity, can convert lactic acid produced by *Streptococcus* metabolism into less acidic propionic acid (Mashima and Nakazawa, 2015). The high-concentration lactic acid swallowed with saliva into the stomach may cause corresponding chemical stimulation to the gastric mucosa, and this stimulation can be alleviated to a certain extent through the aforementioned metabolic transformation, thereby indirectly maintaining gastric acid–base balance.

### 5.3 Immunomodulation and anti-inflammatory effects

Various oral symbiotic bacteria maintain gastric homeostasis by precisely regulating the host immune response. Both live and pasteurised *Lactobacillus crispatus* significantly modulate *H. pylori*-induced inflammation by downregulating mRNA expression of the pro-inflammatory cytokines IL-1β, IL-6, IL-8, and TNF-α, while upregulating the anti-inflammatory cytokines IL-10 and TGF-ß. Specifically, *H. pylori*-induced IL-8 production was markedly suppressed following treatment with either live or pasteurised *Lactobacillus crispatus* (Fakharian et al., 2023). *Lactobacillus* fermentum can downregulate the levels of TNF-α and IL-8, upregulate the expression of IFN-γ and IL-10 in humans, and enhance the host’s ability to resist *H. pylori* infection (Garcia-Castillo et al., 2020). *Lactobacillus rhamnosus GG* (*L. rhamnosus GG*) produces lectin-like proteins 1 and 2, which can inhibit certain pathogens such as Salmonella. By downregulating the expression of various activation and inflammatory markers on monocytes and enhancing the production of interleukin-10, interleukin-12, and tumor necrosis factor-α in macrophages, *L. rhamnosus GG* promotes a type 1 immune response. Through these mechanisms, *L. rhamnosus GG* facilitates recovery from gastritis, gastrointestinal infections and gastric carcinogenesis (Segers and Lebeer, 2014; Capurso, 2019). *L. reuteri* reduces the production of pro-inflammatory factors such as TNF-α and IL-6 by suppressing the NF-κB pathway, and promotes regulatory T cell differentiation to maintain immune homeostasis (Lee et al., 2025). Certain strains, such as DSM 20016, can convert L-histidine into histamine, which then acts via H_2_ receptors to inhibit TNF production by macrophages, thereby exerting an anti-inflammatory effect (Gao et al., 2015).

### 5.4 Reinforcement of the mucosal immune barrier

Specific strains such as *Bifidobacterium longum* and *Bifidobacterium bifidum* can enhance IgA secretion, strengthen the mucosal immune barrier, and block the migration and colonization of oral pathogenic bacteria to the stomach (Kaci et al., 2014; Devine et al., 2015). For patients undergoing gastric cancer resection, intervention with probiotics rich in *Bifidobacterium* can reduce the systemic inflammatory response, promote postoperative recovery of intestinal function and decrease the incidence of complications (Wang et al., 2023).

### 5.5 Improvement of host metabolic health

Certain symbiotic bacteria improve host metabolism by metabolizing dietary fiber to produce short-chain fatty acids (SCFAs). *Prevotella pallens*, as a common symbiotic bacterium in the healthy oral microbiome, can produce SCFAs such as propionic acid by fermenting dietary fiber, exerting anti-inflammatory effects and improving host metabolic health (Han and Wang, 2026). Research by *Han* et al. demonstrated that the severity of gastric symptoms is negatively correlated with the relative abundance of *Prevotella pallens*, suggesting its potential gastric protective role (Han et al., 2019).

## 6 Core mechanisms by which the oral-gastric microbiome axis mediates gastric disease pathogenesis and progression

Under specific conditions, the oral-gastric microbiome axis can also serve as a conduit for pathogens to mediate gastric injury and malignant transformation (**Table 2**).

### 6.1 Disruption of the gastric mucosal barrier

Various oral pathogens compromise the gastric epithelial barrier by degrading cell junction proteins or synergizing with gastric toxins. Degradation of tight junctions: *P. gingivalis*, as a major pathogen of periodontitis, degrades intercellular tight junction proteins by secreting gingipains and can cross the oral mucosal barrier via active migration between epithelial cells or intracellular survival migration (de Jongh et al., 2023). Upon reaching the stomach, its gingipains synergistically act with *H. pylori* toxins to disrupt the gastric epithelial barrier, exacerbating precancerous lesions such as chronic atrophic gastritis and intestinal metaplasia (Muñoz-Medel et al., 2024; Lin et al., 2025). Adhesion molecule interference: *F. nucleatum* binds to E-cadherin on intestinal epithelial cells via FadA adhesins, activating the TLR4/NF-κB signaling pathway; simultaneously, it achieves immune evasion by binding to Gal-GalNAc on host cell surfaces through Fap2 protein (Pignatelli et al., 2023; Zheng et al., 2025). When gastric acid defense mechanisms are compromised (e.g., in chronic atrophic gastritis or prolonged use of proton pump inhibitors), *F. nucleatum* colonizes the stomach and binds to gastric epithelial cells via Fap2 protein, promoting the formation of a tumor microenvironment (Petkevicius et al., 2024).

### 6.2 Activation of pro-oncogenic signaling pathways

Pathogenic bacteria drive the inflammation-carcinogenesis cascade through multiple signaling pathways. The virulence factor CagA of *H. pylori* promotes gastric carcinogenesis by activating the NF-κB-dependent inflammatory pathway, which in turn induces inflammatory cell infiltration, reactive oxygen species-mediated damage, and dysregulated tissue repair responses (Huang et al., 2023). This process aligns with the classic Correa cascade model, wherein *H. pylori* infection initiates and propels a stepwise histopathological progression from normal gastric mucosa through stages of chronic non-atrophic gastritis, atrophic gastritis, intestinal metaplasia, dysplasia, and finally to invasive carcinoma (Zha et al., 2025). Critically, the rising clinical significance of *H. pylori*-negative gastric cancer necessitates a re-evaluation of whether oral-derived bacteria can substitute for *H. pylori* as autonomous triggers of this cascade. Based on current evidence, we propose that these oral microbes occupy distinct stage-specific roles that fall into two functional categories: independent initiators capable of launching the sequence from normal mucosa, and secondary promoters that accelerate progression only after premalignant lesions are established. Within this framework, *Streptococcus anginosus* (*S. anginosus*) promotes gastric inflammation, atrophy, and tumorigenesis in murine models, suggesting an initiator function in *H. pylori*-independent pathways (Fu et al., 2024). By contrast, *F. nucleatum* likely acts as a secondary colonizer that exploits the hypochlorhydric niche created by *H. pylori*-induced atrophy, functioning primarily as a promoter in *H. pylori-*dependent progression (Petkevicius et al., 2024). *P. gingivalis* and *P. intermedia* disrupt epithelial barriers and sustain inflammation (Liang et al., 2024; Muñoz-Medel et al., 2024), but currently lack evidence of independently launching the full Correa sequence; they are therefore classified as stage-restricted promoters operating chiefly from the chronic gastritis-to-dysplasia stages, rather than as primary instigators. *F. nucleatum* and *P. gingivalis* can activate the Wnt/β-catenin signaling pathway through translocation, promote the phosphorylation and translocation of β-catenin to the nucleus, initiate oncogenic transcription programs, and drive the occurrence and development of oral squamous cell carcinoma, gastric cancer, and colorectal cancer (Pignatelli et al., 2023; Wang et al., 2024). *P. gingivalis* activates the TLR2-β-catenin signaling pathway through LPS, promoting the production of reactive oxygen species and inflammatory damage. These signaling events do not uniformly equate to initiating capacity; they serve as mechanistic drivers that amplify malignant transformation once the Correa sequence has been ignited.

### 6.3 Induction of immune evasion and immunosuppressive microenvironment

Pathogenic bacteria promote tumor immune evasion by regulating immune checkpoint molecules. *P. gingivalis* can upregulate PD-L1 expression, induce T cell exhaustion, and promote immune escape in gastric cancer (Muñoz-Medel et al., 2024; Lin et al., 2025). Additionally, H. pylori infection promotes lymphomagenesis by inducing clonal B-cell proliferation and providing persistent antigenic stimulation, leading to the development of mucosa-associated lymphoid tissue (MALT) lymphoma. Eradication therapy leads to histological remission in approximately 70–80% of patients with early-stage gastric MALT lymphoma (Huang et al., 2024).

### 6.4 Generation of pro-inflammatory and pro-carcinogenic microenvironment

Specific bacteria promote carcinogenesis by inducing oxidative stress and DNA damage. Reactive oxygen species and DNA damage: *S. anginosus* induces the production of reactive oxygen species and DNA damage by creating an acidic, hypoxic gastric environment, thereby enhancing the proliferation and invasiveness capacity of gastric epithelial cells (Guo et al., 2024; Xia et al., 2025). The ability of *S. anginosus* to inflict oxidative DNA damage provides a plausible molecular basis for its role as an autonomous initiator, capable of launching the Correa cascade independently of H. pylori by generating the initial genetic lesions that drive the transition from normal mucosa to chronic gastritis.

Release of inflammatory factors: *F. nucleatum* activates the TLR4/NF-κB pathway, leading to the release of pro-inflammatory factors such as IL-6 and TNF-α; *Prevotella intermedia* (*P. intermedia*) is significantly enriched in gastric cancer tissues, with its abundance progressively increasing along the Correa cascade (chronic non-atrophic gastritis → atrophic gastritis → intestinal metaplasia → dysplasia → gastric cancer), and participates in gastric carcinogenesis through mechanisms not yet fully elucidated (Liang et al., 2024; Park et al., 2024).

### 6.5 Metabolic reprogramming promoting tumor progression

Oral-derived microorganisms accelerate tumor progression by altering the metabolic characteristics of the tumor microenvironment. Microenvironment acidification: *Veillonella parvula* and *Streptococcus oralis* form a unique oral-associated microbiota in gastric cancer tissues (particularly in the upper third of the stomach), modifying the tumor microenvironment pH by producing lactic acid and short-chain fatty acids, thereby promoting tumor growth and metastasis, and contributing to spatial microbial heterogeneity with *H. pylori* (Lei et al., 2024). Methionine metabolism and drug resistance induction: *Streptococcus anginosus* promotes metabolic reprogramming in gastric cancer by generating methionine, not only accelerating tumor progression but also inducing drug resistance to tyrosine kinase inhibitors, providing novel microbial intervention targets for gastric cancer treatment (Zhou et al., 2026).

### 6.6 Translocation and colonization via haematogenous or salivary routes

Oral pathogenic bacteria utilize periods of daily bacteremia or compromised gastric acid barrier to migrate into the stomach. *P. intermedia* can enter the human bloodstream through daily bacteremia during daily activities such as chewing and brushing teeth, entering the blood through the damaged periodontal pocket and reaching the gastric mucosa through the bloodstream (Kononen and Gursoy, 2021). Alternatively, it can enter the stomach directly with swallowed saliva, surviving and colonizing under conditions of a damaged gastric acid barrier (Yamazaki and Kamada, 2024; Zhou et al., 2026). Virulence factors of *P. intermedia*, including exopolysaccharides and hemagglutinin, play a key role in facilitating its translocation along the oral-gastric axis (Aitmanaite et al., 2023). *P. intermedia* also expresses hemagglutinin, which binds to sialic acid receptors on gastric epithelial cells, thereby promoting bacterial adhesion and invasion (Park et al., 2021). Notably, virulence varies significantly among different strains of *P. intermedia*. Comparative studies of genomes and phenotypes have shown that differences in exopolysaccharide production ability, hemolytic activity, iron/haemoglobin acquisition ability, and carbohydrate utilization ability lead to significant differences in gastric colonization and pathogenicity among different *P. intermedia* strains (Yamanaka et al., 2011).

### 6.7 Formation of a synergistic carcinogenic microbial network

Various oral-derived bacteria establish synergistic or competitive relationships in the stomach, collectively driving disease progression. Through 16S rRNA sequencing analysis, *Coker* et al. revealed characteristic changes in the gastric microbiome in gastric cancer, showing that *P. intermedia* was significantly enriched in gastric cancer tissues compared to precancerous lesions and healthy controls. Moreover, as the disease progressed, it formed a stronger co-occurrence network with other oral-derived bacteria such as *F. nucleatum*, *Prevotella oris*, and *Catonella morbi* (Dadgar et al., 2023). *Liang* et al. discovered that the abundance of *Prevotella* species and *Helicobacter* species in gastric cancer tissues was negatively correlated, suggesting that *P. intermedia* may play an important role in the occurrence of *H. pylori*-negative related gastric cancer, providing a new perspective for understanding the microbial mechanism of non-*H. pylori*-dependent gastric cancer (Chen et al., 2023; Liang et al., 2024). Furthermore, anaerobic bacteria such as *Peptostreptococcus stomatis* and *Parvimonas micra* have also been confirmed to be in a central position in the gastric cancer ecological network and promote tumorigenesis through metabolic mechanisms that have not been fully elucidated (Coker et al., 2018) (**Figure 2**).

**Figure 2:**
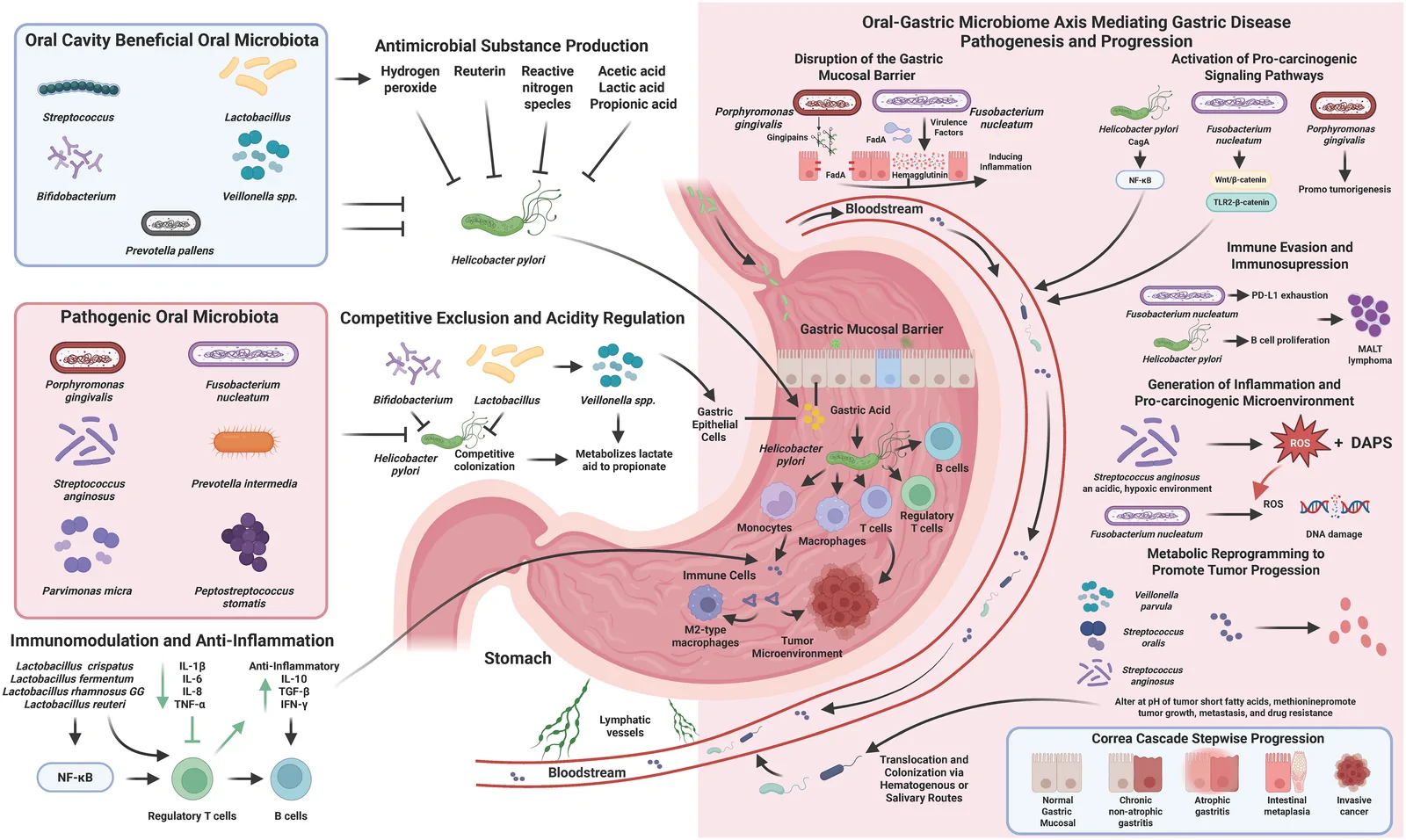
Schematic illustration of the oral–gastric microbiome axis in regulating gastric homeostasis and disease progression. Beneficial oral bacteria (*Streptococcus, Lactobacillus, Bifidobacterium, Veillonella, Prevotella pallens*) produce antimicrobials (H_2_O_2_, reuterin, reactive nitrogen species, SCFAs), regulate pH via lactate-to-propionate conversion, suppress NF-κB, and promote anti-inflammatory responses (IL-10/TGF-β, Treg/B cells) and barrier integrity. Conversely, pathogens (*P. gingivalis*, *F. nucleatum*, *H. pylori*) disrupt the mucosal barrier, induce inflammation, and evade immunity via PD-L1 and B-cell proliferation (MALT lymphoma). *F. nucleatum* also induces ROS/DNA damage and metabolic reprogramming. *H. pylori* participates in competitive exclusion and acidity regulation, and its inflammatory action drives the Correa cascade (normal → chronic gastritis → atrophy → metaplasia → carcinoma). The M2 macrophages, tumour microenvironment, and vascular/lymphatic routes are also shown, illustrating the complex immune-inflammatory-metabolic network.

## 7 Discussion

With advancements in 16S rRNA gene sequencing technology and the exploration of cross-organ microbial axes such as the brain-gut axis, increasing research has revealed that bacterial translocation between the oral cavity and the stomach may exert profound effects on the digestive tract. This review comprehensively delineates the pivotal role of the oral-gastric microbiome axis in mediating gastrointestinal, with a specific focus on elucidating the mechanisms by which key oral microorganisms—including *H. pylori*, *F. nucleatum, P. gingivalis, S. anginosus, and P. intermedia* — translocate, colonize, and promote the pathogenesis of gastric disorders via this axis.

We propose that various pathogenic bacteria and their metabolites within the oral-gastric microbial axis, similar to *H. pylori*, have the potential to serve as non-invasive biomarkers for predicting human diseases. In the future, this approach is expected to evolve into a non-invasive detection method akin to the 13C-urea breath test, enabling convenient screening for gastric diseases. For example, the enrichment of *P. intermedia* in gastric cancer holds significant clinical prognostic value. In terms of diagnostic application, *P. intermedia* can serve as a potential biomarker for gastric cancer. Detecting *P. intermedia* in saliva or gastric mucosa biopsy samples, in combination with other oral bacteria (such as *S. anginosus* and *F. nucleatum*), may assist in the early screening and risk stratification of gastric cancer (Sgamato et al., 2024). Nevertheless, the diagnostic specificity of such oral bacterial biomarkers may be substantially compromised by confounding factors that independently alter oral microbiota composition, including periodontal disease severity, oral hygiene practices, dietary habits, smoking, alcohol consumption, and proton pump inhibitor (PPI) use. Specifically, periodontal disease itself can markedly reshape the oral microbiome and shares multiple risk factors with gastric cancer, thereby potentially confounding the genuine association between microbiota and malignancy. PPIs raise gastric pH and facilitate oral bacterial colonization in the stomach, potentially creating spurious associations with gastric cancer in patients who receive PPIs for dyspeptic symptoms prior to diagnosis. Moreover, behavioural factors such as smoking, alcohol consumption, and dietary patterns exert profound influences on oral microbial community structure; if not adequately controlled, they may readily lead to false-positive or false-negative findings (Parsons et al., 2017; Chen et al., 2019). To improve the clinical applicability of these biomarkers, we advocate the following strategies: (1) multivariate statistical adjustment for the aforementioned covariates in analytical models; (2) multiplexed detection combining oral microbiota profiling with serological markers (e.g., pepsinogen I/II ratio); and (3) integration of multi-omics data (e.g., metagenomics, metabolomics, and host immune parameters) to enhance diagnostic specificity and positive predictive value. Through such systematic adjustments and integrations, the reliability of oral-derived biomarkers in gastric cancer screening can be more robustly assessed in future clinical translation.

On the other hand, eradicating pathogenic bacteria in the oral-gastric axis may serve as a novel therapeutic target for treating upper gastrointestinal diseases. Furthermore, the translocation of microorganisms in the oral-gastric microbial axis may also be the key to breaking through traditional probiotic therapy. Certain bacteria, such as *Streptococcus sanguinis* and *S. mitis* may become novel probiotics by modulating the oral-gastric microecological axis. Therefore, research on the oral-gastric microbiome axis holds significant prospects for clinical application (**Figure 3**).

**Figure 3:**
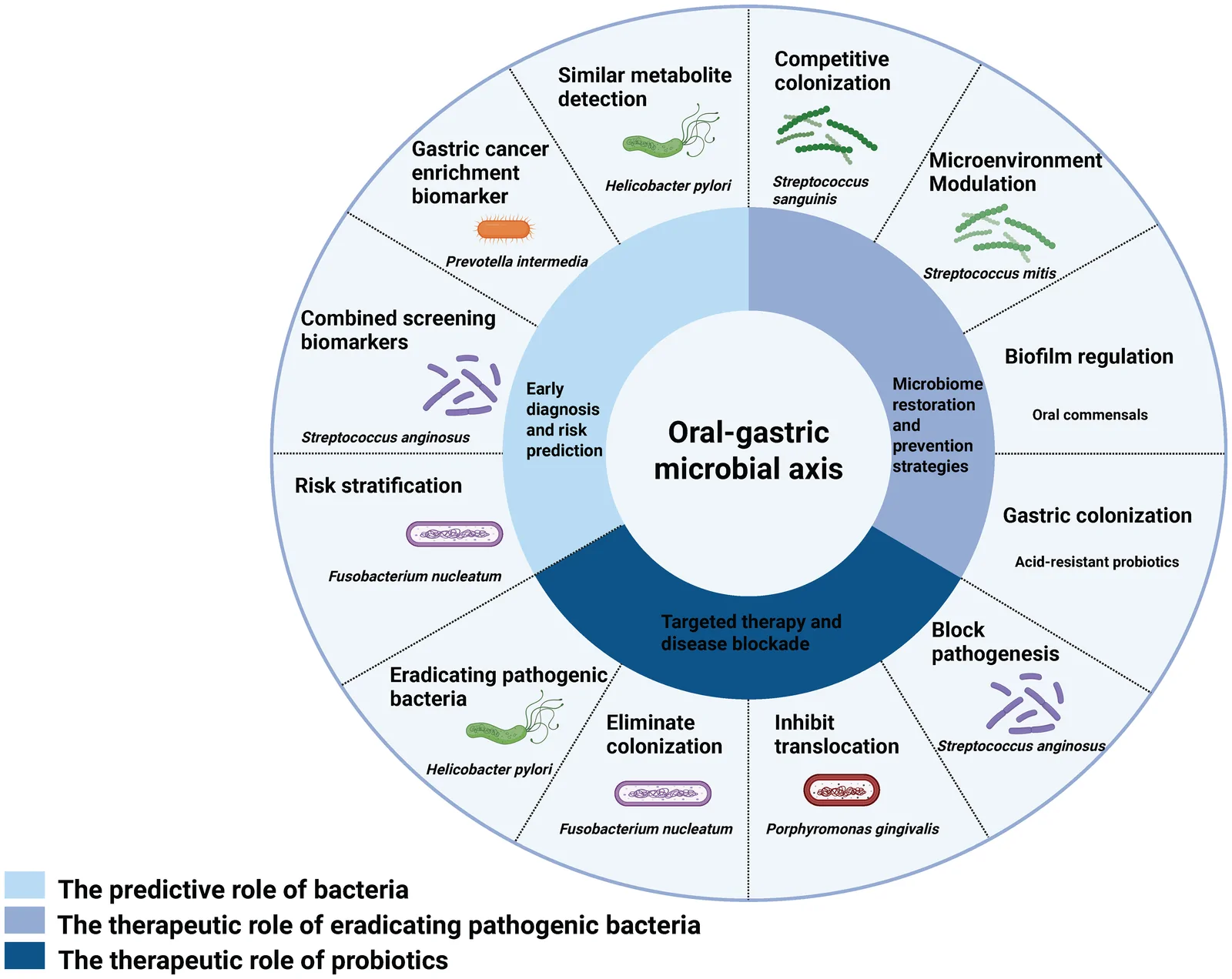
Clinical implications of the oral–gastric microbial axis: a hypothetical framework for diagnosis, targeted therapy, and microbiome restoration. (1) early diagnosis and risk prediction using bacterial biomarkers; (2) targeted therapy by eradicating pathogenic bacteria via elimination of colonization, inhibition of translocation, and blockade of pathogenesis; and (3) microbiome restoration and prevention strategies, including competitive colonization, microenvironment modulation, biofilm regulation, and use of acid-resistant probiotics.

Nevertheless, current research faces several limitations and challenges. Firstly, investigations into the oral-gastric microbiome axis predominantly rely on cross-sectional or retrospective analyses, lacking large-scale prospective cohort studies to establish causal relationships between specific microorganisms and gastric disease. Secondly, although animal models have confirmed that individual oral bacterial species can accelerate gastric lesion progression, the complexity of the human gastric microbiome surpasses that of experimental models, necessitating further exploration of synergistic or antagonistic interactions among multiple oral bacteria.

In the future, novel technology and standardized metrics are needed to identify potential pathogenetic microbes and the core microbiota, location, interaction, effect, richness and colonization. In addition, current research on the oral-gastric biological axis is mostly confined to bacteria. However, as indispensable components of this axis, the dynamic changes of non-bacterial microorganisms such as fungi and viruses are equally crucial and urgently need in-depth exploration. It is essential to comprehensively incorporate the oral-gastric microbiome into the human microbiome research initiative and elucidate the intricate ecological interactions, structural relationships, physiological connections, and metabolic cross-species dynamics within it.
